# Increasing emergency number utilisation is not driven by low-acuity calls: an observational study of 1.5 million emergency calls (2018–2021) from Berlin

**DOI:** 10.1186/s12916-023-02879-7

**Published:** 2023-05-16

**Authors:** David Herr, Sangeeta Bhatia, Florian Breuer, Stefan Poloczek, Christopher Pommerenke, Janosch Dahmen

**Affiliations:** 1 Faculty of Medicine, School of Public Health, Imperial College London, South Kensington Campus, London, SW7 2 AZ UK; 2 MRC Centre for Global Infectious Disease Analysis, School of Public Health, Jameel Institute, Imperial College London, London, UK; 3 Emergency Medical Services Director, Rhine-Berg District, Office for Fire Protection and Emergency Medical Service, Bergisch Gladbach, Germany; 4 Office of the Medical Director, Emergency Medical Services, Berlin, Germany; 5 Faculty of Health, Department of Medicine, Witten/Herdecke University, Witten, Germany

**Keywords:** Emergency Medical Service, Emergency calls, Ambulance, 112, 999, Low-acuity, Overload, Emergency medicine

## Abstract

**Background:**

The Emergency Medical Service (EMS) in Germany is increasingly challenged by strongly rising demand. Speculations about a greater utilisation for minor cases have led to intensive media coverage, but empirical evidence is lacking. We investigated the development of low-acuity calls from 2018 to 2021 in the federal state of Berlin and its correlations with sociodemographic characteristics.

**Methods:**

We analysed over 1.5 million call documentations including medical dispatch codes, age, location and time using descriptive and inferential statistics and multivariate binary logistic regression. We defined a code list to classify low-acuity calls and merged the dataset with sociodemographic indicators and data on population density.

**Results:**

The number of emergency calls (phone number 112 in Germany) increased by 9.1% from 2018 to 2021; however, the proportion of low-acuity calls did not increase. The regression model shows higher odds of low-acuity for young to medium age groups (especially for age 0–9, OR 1.50 [95% CI 1.45–1.55]; age 10–19, OR 1.77 [95% CI 1.71–1.83]; age 20–29, OR 1.64 [95% CI 1.59–1.68] and age 30–39, OR 1.40 [95% CI 1.37–1.44]; *p* < 0.001, reference group 80–89) and for females (OR 1.12 [95% CI 1.1–1.13], *p* < 0.001). Odds were slightly higher for calls from a neighbourhood with lower social status (OR 1.01 per index unit increase [95% CI 1.0–1.01], *p* < 0.05) and at the weekend (OR 1.02 [95% CI 1.0–1.04, *p* < 0.05]). No significant association of the call volume with population density was detected.

**Conclusions:**

This analysis provides valuable new insights into pre-hospital emergency care. Low-acuity calls were not the primary driver of increased EMS utilisation in Berlin. Younger age is the strongest predictor for low-acuity calls in the model. The association with female gender is significant, while socially deprived neighbourhoods play a minor role. No statistically significant differences in call volume between densely and less densely populated regions were detected. The results can inform the EMS in future resource planning.

## Background

In recent months and years, intensive debate including regional and national media coverage [1, 2] occurred about increasing pressure on the Emergency Medical Service (EMS) throughout Germany and specifically in Berlin. This includes the question whether the rising demand [3, 4] is primarily due to minor cases, which should not be handled by the EMS. Some speculated about an increasingly demanding patient attitude up to the point of an ‘all-inclusive mentality’, meaning that citizens expect immediate maximum care by the EMS even for minor problems, regardless of financial and opportunity costs [5]. However, there has been very little evidence to prove or reject this hypothesis. Low-acuity calls to the EMS have been investigated in several studies worldwide [6–13], but most data is several years old and often from non-European settings like the United States of America (US). Very few studies investigate German data with this particular focus [14–16], which results in a significant research gap.

There is more evidence for emergency departments (EDs) of hospitals. Low-acuity ED cases have been shown to increase, contributing to over-crowding and inefficient resource use [17, 18].

When investigating EMS utilisation, it is crucial to consider the socioeconomic context, especially as the sociodemographic structure of Berlin is very heterogeneous [19]. Social determinants are associated with disease prevalence and health care utilisation [20], and the burden of disease strongly correlates with age [21]. People in socially deprived areas and migrants have specific health care needs [22]. Migrants and foreigners might approach the health system and the 112 service differently [23]. There can be language barriers and a lower health literacy [22, 24] which complicate identifying the best-suited level of care. For other German regions, researchers have also pointed at differences in EMS utilisation depending on population density (‘urban’ vs. ‘rural’ areas) [14, 16].

## Methods

### Aim

This study aims to investigate whether the proportion of low-acuity calls to the EMS Berlin has increased from 2018 to 2021 and whether their frequency correlates with sociodemographic characteristics of the districts and smaller geographical areas, like a lower social status of the population and a higher proportion of poor elderly people, migrants and foreigners who have less access to alternative health care resources. By shedding light on the regional differences and temporal development of utilisation and on predictors, the study also aims to contribute to EMS resource allocation and to the identification of possible target groups for specific health education.

### Setting

The German health system includes a strong ambulatory care sector with general practitioners and also specialists in own medical practices, however with limited capacity outside regular opening hours [25]. The emergency infrastructure is generally free of charge without co-payments. About 90% of people in Germany are statutory health insured, the others have—with some exceptions of specific coverage—substitutive private health insurance [25]. The EMS in Berlin, run by the fire department, is responsible for taking emergency calls (phone number 112), dispatching ambulances, and transporting patients with acute conditions to hospitals [26]. Besides EMS and EDs, an On-Call Medical Service (OCMS) of the association of statutory-health insurance physicians (phone number 116 117) exists, which is meant to replace regular medical practices outside of regular opening hours. The OCMS typically does home visits, but within a time limit of up to a couple of hours. So, it is not there for the very acute cases. In Berlin, about hundred low-acuity EMS calls are being transferred to the OCMS per day [26].

### Design

**Fig. 1:**
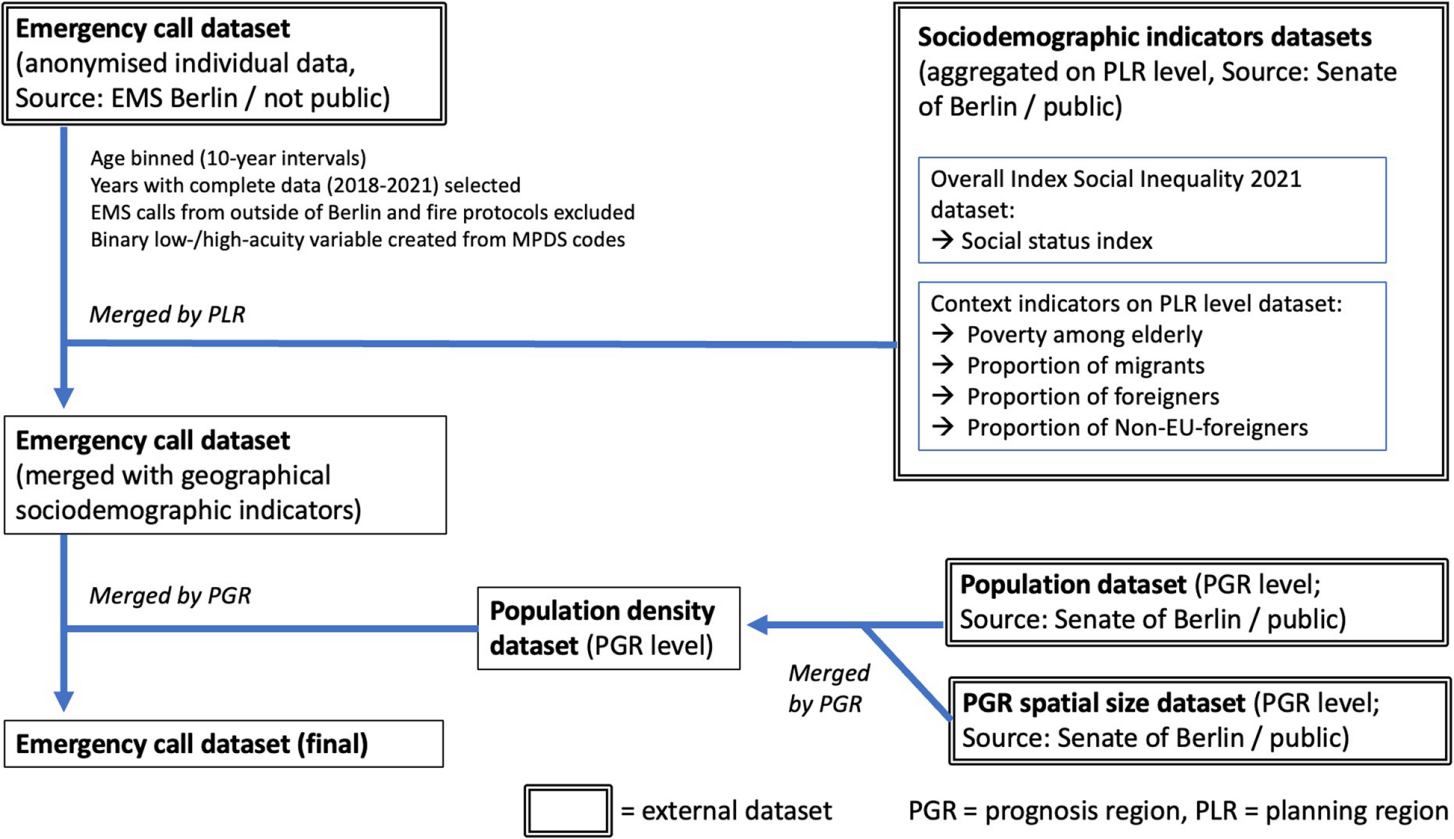
Data flow chart. Source: Own illustration

The study uses a cross-sectional observational design. We performed analyses of electronic dispatcher documentations including merged, region-related sociodemographic indicators (Fig. 1).

The emergency call dataset was provided by the Office of the EMS medical director and covers September 2017 to July 2022. Only data from full years (2018–2021) was included in the analyses to avoid bias due to seasonal effects. Calls from outside of Berlin and fire protocols were excluded. Transferrals to the OCMS were included. Excerpts of the original data have been used for earlier utilisation analyses, like on frequent users in 2018 [15]. The fire department anonymised the dataset by deleting patients’ names, transforming the exact emergency locations into geographical areas (life-world-oriented regions, LORs) and coarsening date and time. Berlin’s LOR system classifies geographical areas in four hierarchical steps: district (*n* = 12), prognosis region (PGR, *n* = 58), district region (BZR, *n* = 143) and planning region (PLR, *n* = 542).

Medical Priority Dispatch System (MPDS, Salt Lake City, UT, USA) [27] is used in a minority of dispatch centres in Germany, albeit increasingly [28], and is widespread in the US and the United Kingdom (UK) [11]. The EMS Berlin has been using MPDS for over 10 years. Each call gets assigned a code depending on chief complaint and context. Each code (e.g. ‘29D2m’) consists of a protocol (e.g. ‘29’ for traffic accident), a category (Omega is the lowest, followed by Alpha, Bravo, Charlie, Delta and Echo), a sub-determinant (e.g. ‘2’ for ‘dangerous accident mechanism’) and a suffix (e.g. ‘m’ for ‘car against pedestrian’). No consensus on which calls to classify as low-acuity exists [12, 13, 29, 30]. We defined an own code list in consultation with experts of the Berlin EMS, based on international literature—e. g. from the UK [10], US [29] and Denmark [31, 32]—and local dispatch guidelines. We defined low-acuity as the following: all Omega codes, all Alpha codes except protocol 12 (seizures), and all ##B00# and ##C00# codes (upcodings from Omega/Alpha due to OCMS unavailability or other contextual factors) except protocol 12.

We merged publicly available additional datasets with sociodemographic indicators (*Overall Index Social Inequality 2021* [33] and *Context Indicators 2021* [34], CC-BY-3.0 license) on PLR level (Fig. 1). We explored variables on social status (index of unemployment and social benefit recipients among adults and minors), poverty among elderly (proportion of social benefit recipients above retirement age) and the proportions of migrants and foreigners (Table 1).

**Table 1:** Region-related sociodemographic indicators explored for regression analysis, merged on PLR level

**Variable**	**Type and calculation**	**Latest available measurement date**	**Data source**^a^
Social status index (SOCIALSTATUS)	Continuous index variable, ranging from -1.5 (highest social status) to 4 (lowest social status) Three-component index of a) Unemployment and b) Social benefit recipients among adults and c) Social benefit recipients among minors	31 December 2020	*Overall Index Social Inequality 2021 (Status/Dynamic) on LOR level*
Poverty among elderly (ELDERLYPOVERTY)	Continuous variable, 0–100%. Proportion of social benefit recipients among inhabitants above the regular retirement age	31 December 2018^b^	*Context Indicators on PLR level, LOR boundaries of 2021; indicator K03*
Proportion of migrants (MIGRANTS)	Continuous variable, 0–100%. Proportion of migrants among all inhabitants	31 December 2018^b^	*Context Indicators on PLR level, LOR boundaries of 2021; indicator K05*
Proportion of foreigners (FOREIGNERS)	Continuous variable, 0–100%. Proportion of foreigners among all inhabitants	31 December 2018^b^	*Context Indicators on PLR level, LOR boundaries of 2021; indicator K16*
Proportion of non-EU-foreigners (NON-EU-FOREIGNERS)	Continuous variable, 0–100%. Proportion of non-EU-foreigners among all foreigners	31 December 2018^b^	*Context Indicators on PLR level, LOR boundaries of 2021; indicator K17*

^a^ All sociodemographic data sources are available from the Senate Department for Urban Development, Building and Housing Berlin/Office for Statistics Berlin-Brandenburg

^b^ The geographical classifier in datasets with data from 2018 was adapted by the government according to the modification of the LOR system in 2020

We classified population density on PGR level, making a cut-off between ‘*densely populated*’ and ‘*less densely populated*’ PGRs at 3000 inhabitants per square kilometre. This concept is similar to ‘rurality’ in some other studies [14, 16]. However, we avoid the term *rural region* because no regions with very low population density exist in Berlin. To calculate population density, we merged two other datasets of the Senate including population of PGRs [35] and geographical size of PGRs [36].

### Data analysis

Missing data (NAs) was generally handled with pairwise deletion for descriptive analyses, on the assumption that values are missing-at-random. However, Figs. 3, 4 and 5 include missing values to provide a complete picture of the overall number of calls and the temporal development of NAs. We applied listwise deletion of NAs for regression analysis. This includes all calls from PLRs with < 1000 inhabitants (1% of PLRs), because the sociodemographic datasets do not include values for these PLRs for reasons of data protection.

We binned age in 10-year intervals to account for the observation of peaks at round ages, which is likely due to third-person callers guessing the patient’s age, e.g. in case of a patient’s unconsciousness. We analysed time trends using descriptive statistics. Pearson’s chi-squared tests and *t*-tests were used for group comparisons with categorical and quantitative dependent variables. We performed multivariate logistic regression on the 2021 individual data, which uses the same updated LOR system as the sociodemographic indicators, with low-acuity as binary outcome. To check for collinearity among independent variables, we computed a correlation matrix before building the regression model and used variable inflation factor (VIF) analysis upon the final model. Within forward selection of variables, we did not include predictors if collinearity was high (> 0.5) or if they did not noticeably improve the model, to avoid overfitting. We tested the model’s predictive performance with Hosmer–Lemeshow and McFadden’s *R*^2^ tests. All statistical analyses were performed using RStudio (PBC, 2022.07.1 Build 554). All results are considered exploratory, not confirmatory. We built the regression model performing forward stepwise selection of variables (see the “Results” section).

## Results

Table 2 shows main characteristics of the sample. The average patient age was 54 years, with 47% females, 52% males and 1% unknown gender. 12.6% of all calls had missing data of at least one variable. MPDS protocols had no missing data, but MPDS categories had, more so in earlier years (14.1% in 2018 compared to 0.4% in 2021).

**Table 2:** Basic characteristics of the emergency call data sample from 2018 to 2021

**Variable**	**Year**					**Missing data**
	**2018**	**2019**	**2020**	**2021**	**Total (2018–2021)**	**Total (2018–2021)**
*n*	394,736	405,277	414,435	430,674	1,645,122	207,193 (12.6%)^a^
Age (mean [SD])	53.0 [26.0]	53.9 [25.3]	54.8 [25.0]	54.5 [25.5]	54.1 [25.5]	74,585 (4.5%)
Gender						22,102 (1.3%)
*Female*	*47.3%*	*47.0%*	*46.6%*	*47.2%*	*47.0%*	
*Male*	*51.4%*	*51.8%*	*52.1%*	*51.3%*	*51.6%*	
*NA*	*1.3%*	*1.3%*	*1.3%*	*1.5%*	*1.3%*	
Weekday (mean [SD])	4 [2]	4 [2]	4 [2]	4 [2]	4 [2]	0
Hour (mean [SD])	12.7 [6.3]	12.8 [6.3]	12.9 [6.2]	12.8 [6.3]	12.8 [6.3]	0
MPDS protocol						0
MPDS category						157,055 (9.5%)
District						15 (< 0.1%)
*Charlottenburg-Wilmersdorf*	*9.5%*	*9.5%*	*9.1%*	*9.0%*	*9.2%*	
*Friedrichshain-Kreuzberg*	*7.9%*	*8.1%*	*7.5%*	*7.4%*	*7.7%*	
*Lichtenberg*	*7.7%*	*7.9%*	*8.1%*	*8.2%*	*8.0%*	
*Marzahn-Hellersdorf*	*7.1%*	*6.9%*	*7.2%*	*7.4%*	*7.1%*	
*Mitte*	*12.7%*	*12.5%*	*12.0%*	*12.1%*	*12.3%*	
*Neukölln*	*9.2%*	*9.0%*	*9.3%*	*9.0%*	*9.1%*	
*Pankow*	*8.6%*	*8.7%*	*8.8%*	*9.0%*	*8.8%*	
*Reinickendorf*	*7.4%*	*7.4%*	*7.5%*	*7.6%*	*7.5%*	
*Spandau*	*7.0%*	*6.9%*	*7.1%*	*6.9%*	*7.0%*	
*Steglitz-Zehlendorf*	*6.6%*	*6.8%*	*6.9%*	*7.1%*	*6.9%*	
*Tempelhof-Schöneberg*	*8.8%*	*8.8%*	*8.8%*	*8.7%*	*8.7%*	
*Treptow-Köpenick*	*7.4%*	*7.4%*	*7.6%*	*7.6%*	*7.5%*	

The MPDS suffix was not used for any analyses and is therefore not included in the table

^a^ Missing data of at least one of the variables presented in the table

**Fig. 2:**
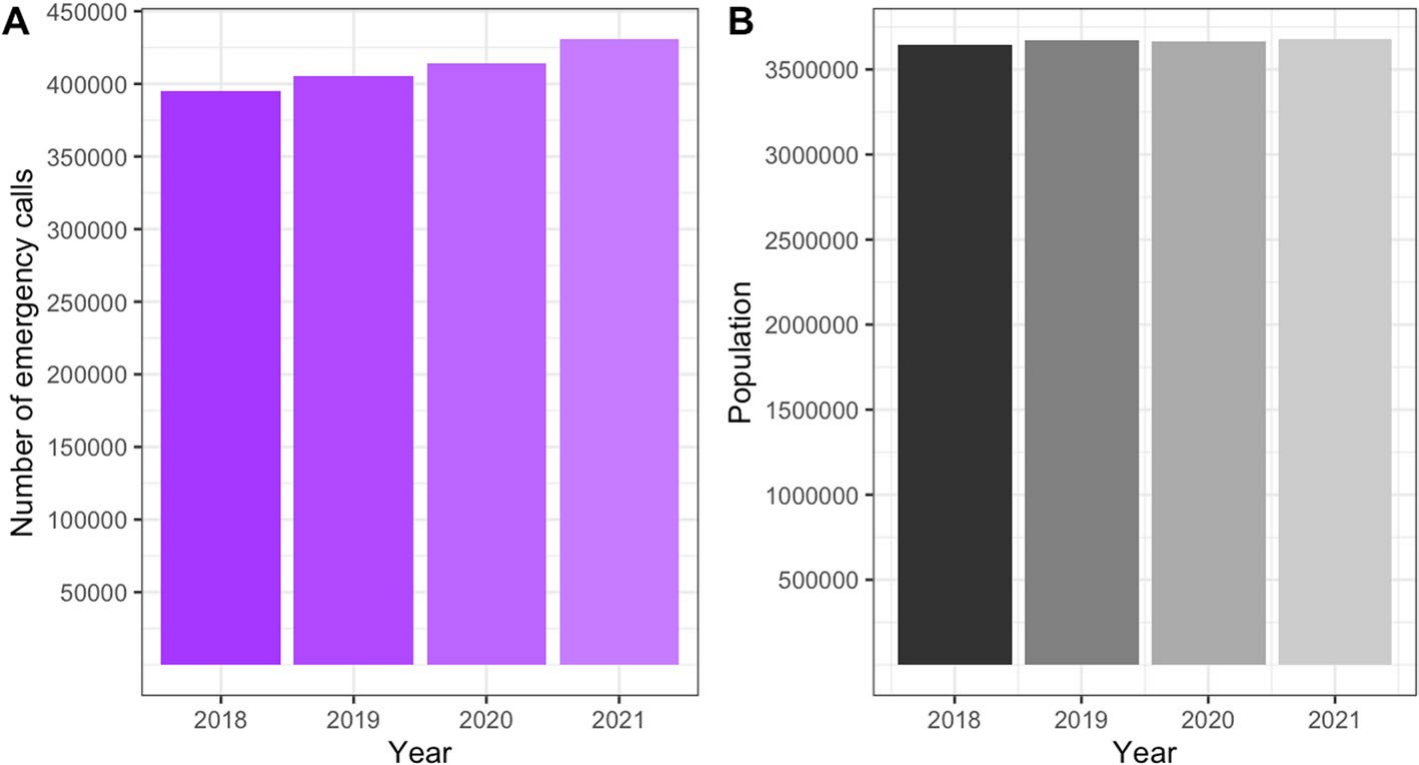
Temporal development from 2018 to 2021 of **A** the number of emergency calls and **B** the population of Berlin. Source: **A** Data of the EMS Berlin and **B** data of the Office for Statistics Berlin-Brandenburg

The total number of 112 calls increased by 9.1% from 394,736 calls in 2018 to 430,674 calls in 2021 (Fig. 2). Meanwhile, the population increased to a far lesser extent, by 0.9% from 3,644,826 to 3,677,472 [37]. More calls concerned male (52.3% in 2021) than female patients.

When not considering calls with unspecified acuity, the proportion of low-acuity calls decreased from 34.7% in 2018 to 30.0% in 2021, with higher proportions among females (from 37 to 32%) than among males (from 33 to 29%). The proportion of more severe calls (especially Bravo, followed by Charlie and Delta, data not shown) increased. Among the low-acuity calls of 2021, Omega codes accounted for only 1.5%, Alpha for 79%, the rest were B00/C00 upcodings.

**Fig. 3:**
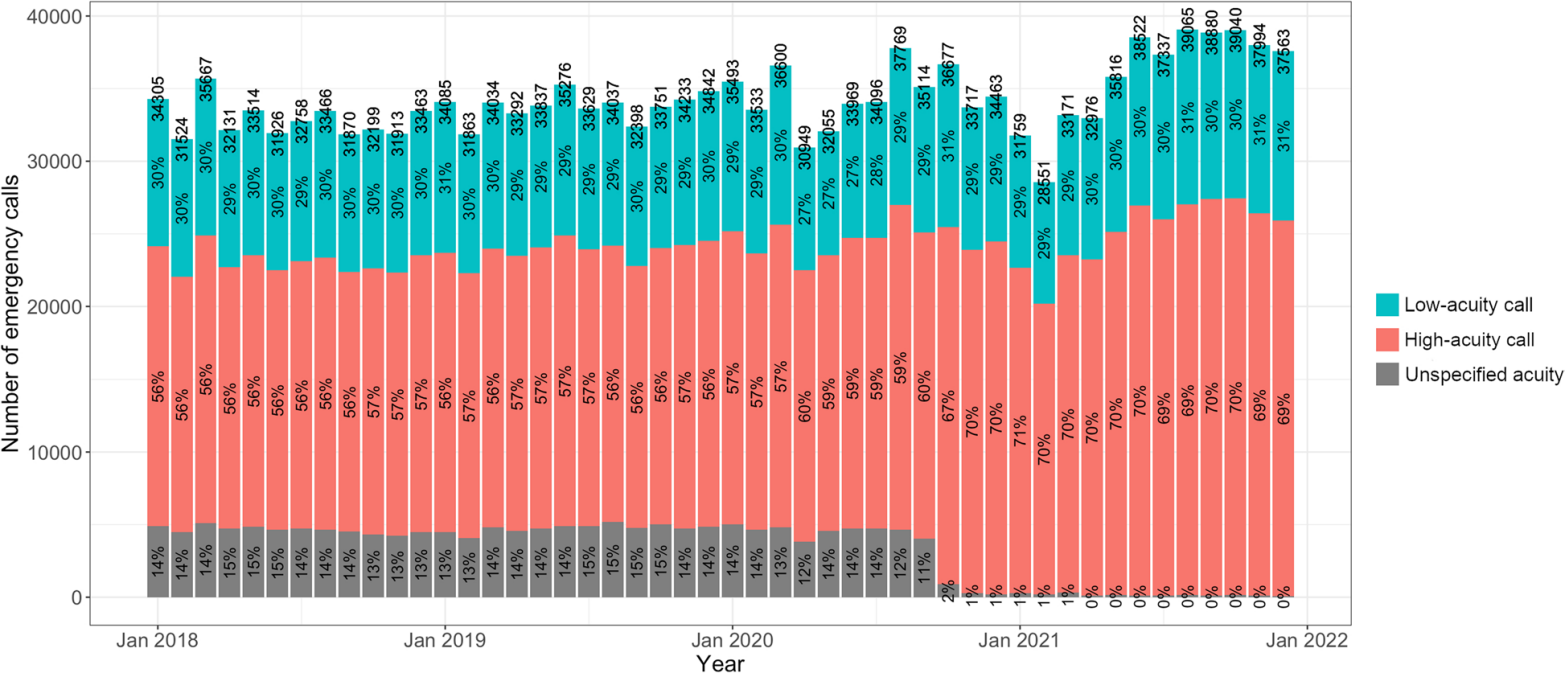
Temporal development of low-acuity and high-acuity emergency calls from January 2018 to December 2021. Source: Data of the EMS Berlin

A month-by-month illustration of the low- and high-acuity calls—including calls with unspecified acuity due to protocol aborts (Fig. 3)—demonstrates the increasing number of overall emergency calls. There are notable temporary decreases in April 2020 (accompanied by a slight decrease of the low-acuity proportion) and in February 2021 (constant low-acuity call proportion). The number of calls with unspecified acuity is persistently between 14 and 12% until August 2020 and is then reduced to 1% within 4 months and, later, to zero (increasing protocol adherence). Within the same 4-month period of July to November 2020, the percentage of documented high-acuity calls increases from 58.5 to 70%, and the percentage of documented low-acuity calls increases to a far lesser extent, from 27.5 to 29%.

**Fig. 4:**
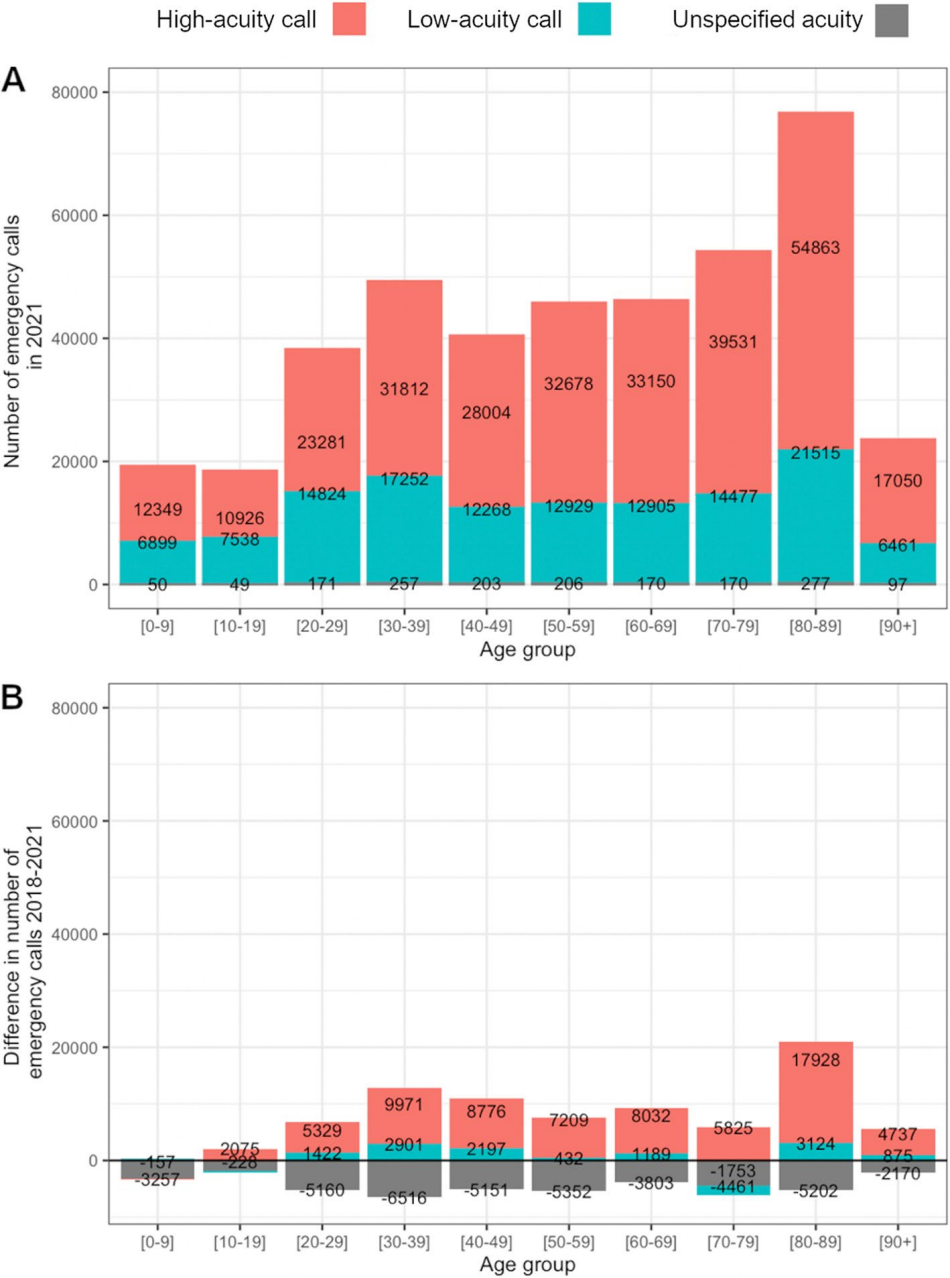
**A** Number of low-acuity and high-acuity calls per age group in 2021 and **B** difference between 2018 and 2021. NAs of MPDS category have been included to correctly plot the total call numbers and to show the increasing protocol adherence (decrease of NAs) over time. Source: Data of the EMS Berlin

The number of emergency calls and the low-acuity proportion differed between age groups. In the tendency, the overall number was higher in older age groups, while the proportion of low-acuity calls was lower (Fig. 4). For example, the largest age group 80–89 included 76,655 calls in 2021, compared to 18,513 calls in the age group 10–19.

A deeper analysis of the 2021 data shows the following: the low-acuity proportion was highest in age group 10–19 (41%), becoming smaller in older age with a minimum in age groups 70–79 and 90 + (*p* < 0.001). It was very similar between weekdays, slightly higher for Sundays (31% vs. 30% for all other weekdays). Nevertheless, the difference was significant for weekend as a binary variable (chi-squared test: *p* < 0.05). There was no statistically significant association with the time of day (chi-squared test: *p* = 0.02).

**Fig. 5:**
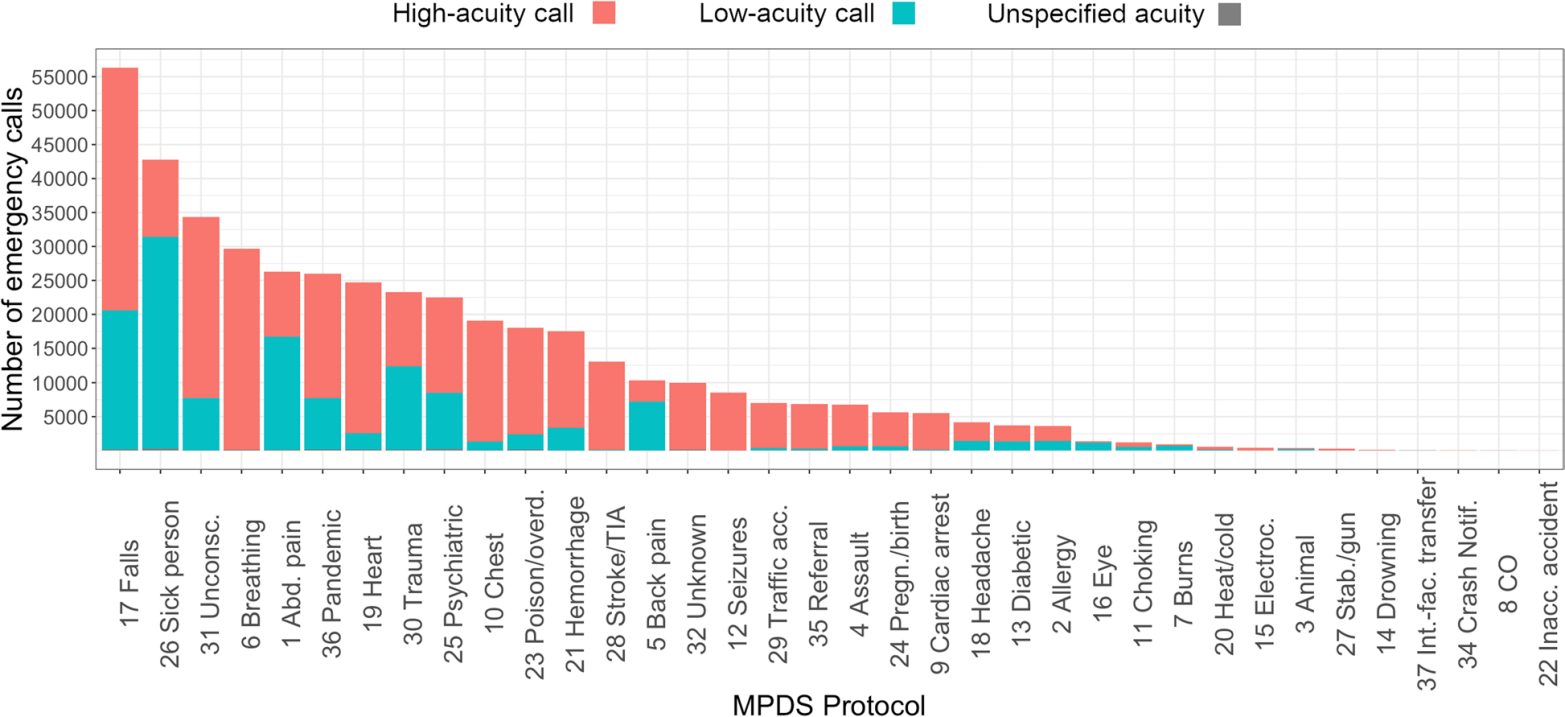
Distribution of MPDS protocols (first element of the MPDS code, chief complaint) and proportion of low-acuity calls in 2021. Source: Data of the EMS Berlin. Protocols: 1: Abdominal pain, 2: Allergies*/*envenomations, 3: Animal bites*/*attacks, 4: Assault*/*sexual assault, 5: Back pain, 6: Breathing problems, 7: Burns*/*explosions, 8: Carbon Monoxide*/*inhalation, 9: Cardiac or respiratory arrest*/*death, 10: Chest pain, 11: Choking, 12: Convulsions*/*seizures, 13: Diabetic problems, 14: Drowning*/*diving, 15: Electrocution*/*lightning, 16: Eye problems, 17: Falls, 18: Headache, 19: Heart problems, 20: Heat*/*cold exposure, 21: Hemorrhage*/*lacerations, 22: Inaccessible incident, 23: Overdose*/*poisoning, 24: Pregnancy*/*childbirth*/*miscarriage, 25: Psychiatric*/*suicide attempt, 26: Sick person, 27: Stab*/*gunshot*/*penetrating trauma, 28: Stroke*/*transient ischemic attack, 29: Traffic incidents, 30: Traumatic injuries, 31: Unconscious*/*fainting, 32: Unknown problem, 33: Inter-facility transfer*/*palliative care, 34: Automatic crash notification, 35: Health-care practitioner referral, 36: Pandemic*/*epidemic*/*outbreak, 37: Inter-facility transfer specific to medically trained callers

The low-acuity proportion differs strongly between MPDS protocols (Fig. 5). The protocols for *breathing problems*, *seizures* and *helpless persons* can per definition not include low-acuity calls, because either the MPDS system contains only Charlie to Echo categories [6, 32] or it was excluded by the low-code definition of this study [12]. Some protocols concern nearly always higher-acuity calls, e.g. for *chest pain*, *heart problem*, *intoxication*, *pregnancy/birth* and *stroke.* Others consist of a majority of low-acuity calls*: abdominal pain*, *back pain*, *sick person* and *injury.* The protocols regarding *fall, psychiatric problem*, *fainting* and *pandemic* consist of a majority of higher-acuity calls but nevertheless make up for a relevant number of low-codes because of the protocols’ overall frequencies.

Some specific MPDS codes stand out in terms of frequency: The most common low-code in 2021 is 01A01 (abdominal pain without complications, *n* > 15,000), followed by 30A02 (injury of a harmless body part, *n* > 9500), 26B00 (upcoded to Bravo, sick person without emergency symptoms, *n* > 8000), 26A07 (weakness without emergency symptoms, *n* > 6500), 26A08 (pain without emergency symptoms, *n* > 6500), 17A02 (fall on non-dangerous body part, *n* > 6000) and 31A01 (fainting but responsive with age ≥ 35, *n* > 5000).

### Effects of the SARS-CoV-2 pandemic

Stay-at-home orders (‘lockdowns’) in Germany due to the SARS-CoV-2 pandemic were not uniform regionally and regarding their strictness. A strict nationwide lockdown took place from 22 March to 4 May 2020, a second relatively strict one from mid-December 2020 until the end of January 2021. Less strict lockdowns took place in November 2020 and June 2021, plus regionally in different time periods. The month-by-month analysis (Fig. 3) shows a drop (− 15%) of overall emergency calls in the ‘lockdown month’ April 2020 compared to the month before, combined with a decrease of the low-acuity proportion from 30% to 27%. The effect was compensated within 4 months. In January 2021 (but even more in February 2021), there is also a, albeit smaller, drop in overall emergency calls, compensated within 2 months, this time with an unchanged low-acuity proportion. It is possible, although speculative, that these changes in utilisation (especially regarding less urgent cases) reflect either a different incidence of emergencies and/or a different behaviour of patients, because the fear of infection may prevent patients from utilising services. This would match observations in many countries like a reduction of ED visits and hospital admissions [38, 39]. There is no evidence in our data that the described effects would have led to longer-term changes in EMS utilisation.

### Geographical analysis

**Fig. 6:**
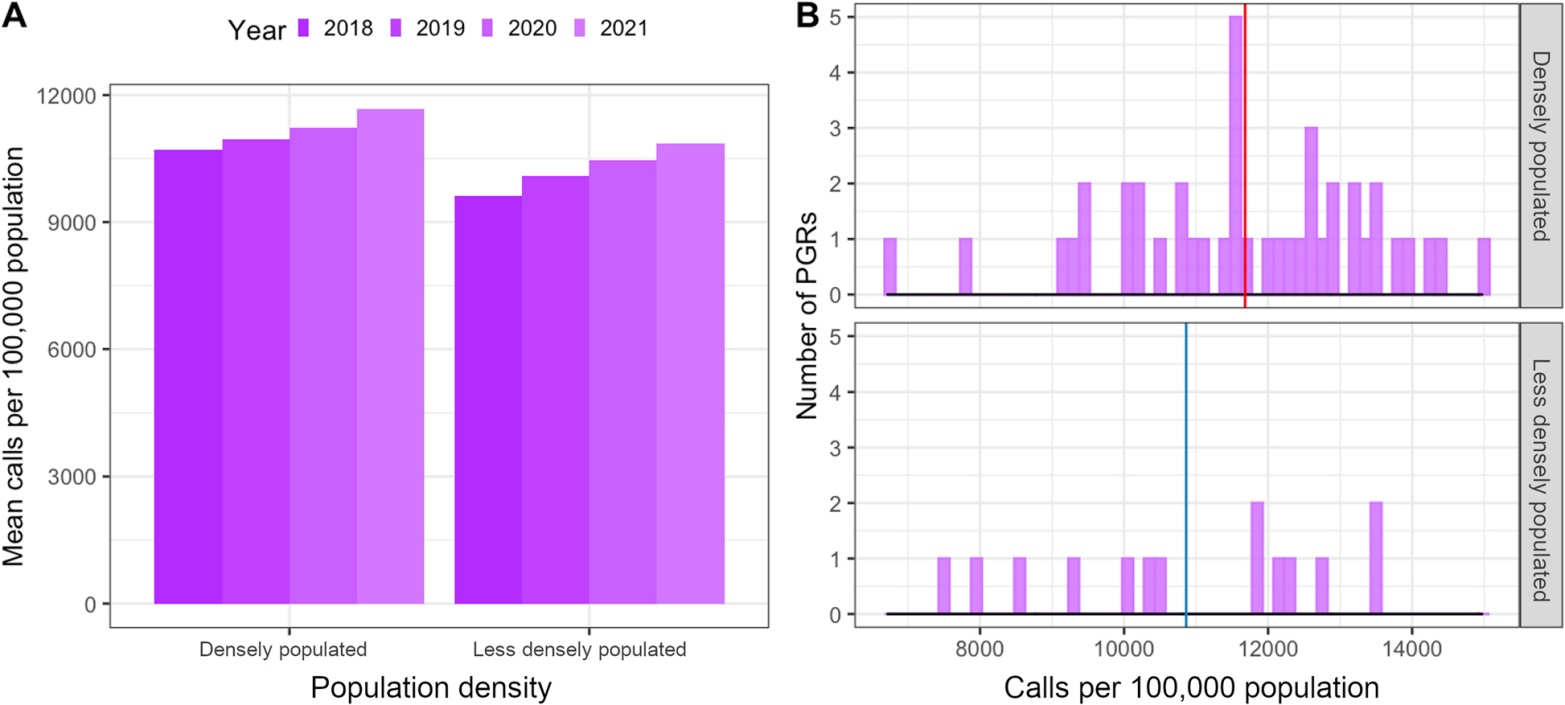
Comparison of densely populated (> 3000 inhabitants per km^2^) and less densely populated PGRs regarding mean number of calls in 2021. **A** Mean of emergency calls per 100,000 inhabitants in a PGR in the years 2018–2021. **B** Histogram plotting all PGRs depending on the rate of emergency calls per 100,000 inhabitants in 2021. Vertical lines represent the means for densely populated (red) and less densely populated (blue) PGRs. The differences between densely and less densely populated PGRs were not statistically significant. Source: Data of the EMS Berlin and of the Senate of Berlin

Densely populated (> 3000 inhabitants per km^2^) and less densely populated PGRs show similar call increase patterns from 2018 to 2021 (Fig. 6), albeit from a higher starting level in densely populated regions. The group differences were not statistically significant in any year (unpaired *t*-test for 2021: *p* = 0.19).

**Fig. 7:**
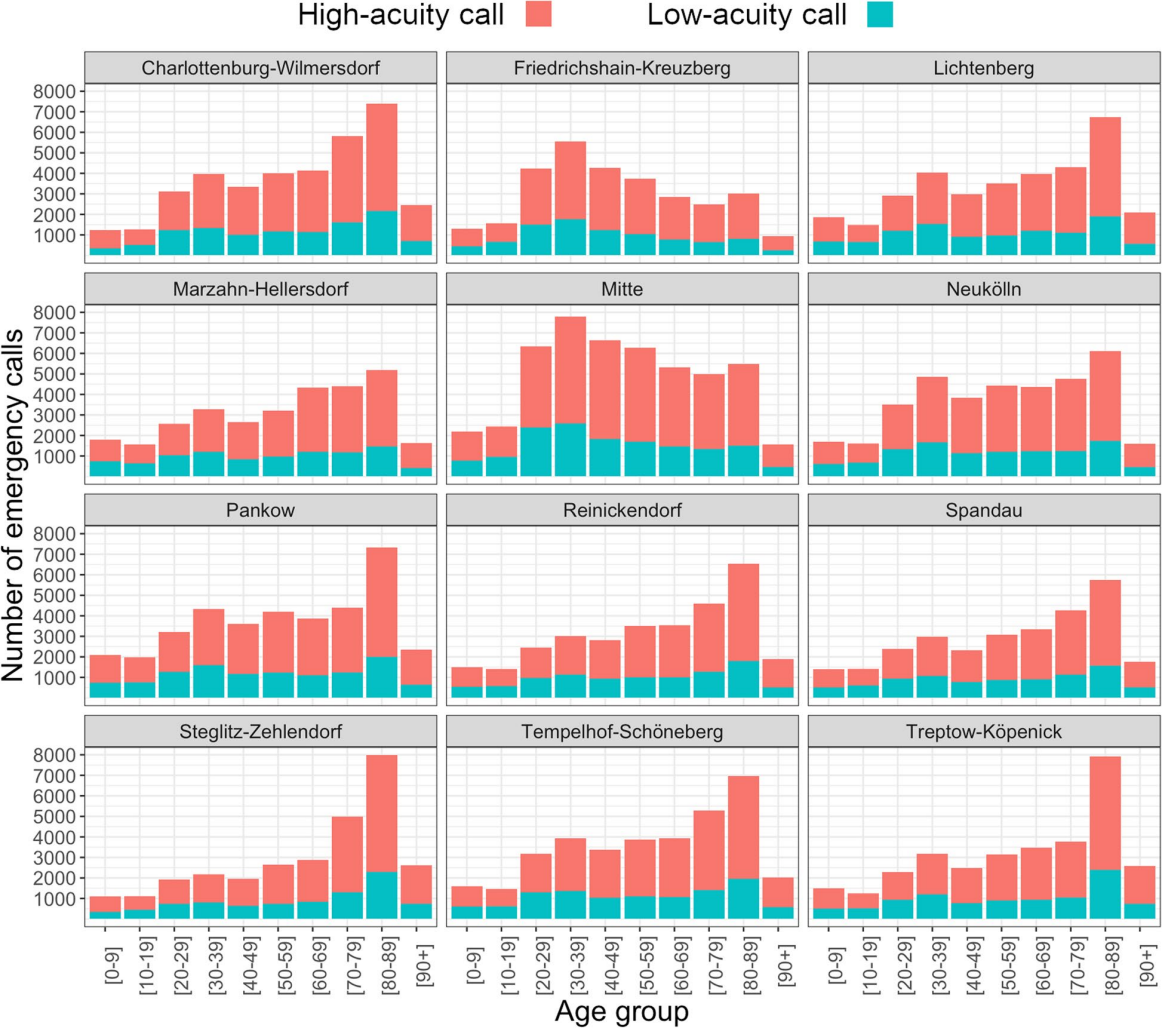
Age distribution of low- and high-code emergency calls per district of Berlin and per acuity in 2021. Source: Data of the EMS Berlin

The districts show different numbers of calls per age group (Fig. 7). For example, a high call volume concerns elderly people in Steglitz-Zehlendorf and Treptow-Köpenick, while calls concerning younger adults are very common in Mitte and Friedrichshain-Kreuzberg.

The overall low-acuity proportion differs only little, but statistically significantly between districts (*p* < 0.001). The highest proportion can be observed in Treptow-Köpenick, Marzahn-Hellersdorf, Lichtenberg and Pankow (31%), the lowest in Charlottenburg-Wilmersdorf, Friedrichshain-Kreuzberg and Mitte (29%). It was 30% in the other districts.

### Logistic regression

AGEGROUP, GENDER, SOCIALSTATUS of the neighbourhood (Table 1) and WEEKEND (binary variable) significantly improved the model during stepwise selection of variables. The correlation matrix showed high collinearity (> 0.5) of SOCIALSTATUS with ELDERLYPOVERTY, FOREIGNERS and MIGRANTS, and even higher between MIGRANTS and FOREIGNERS (> 0.9) and between ELDERLYPOVERTY and FOREIGNERS or MIGRANTS (> 0.7). Furthermore, the odds ratios (ORs) of each MIGRANTS and FOREIGNERS, as additional predictor besides SOCIALSTATUS, were negligible (1.0, 95%-confidence interval (CI): 1.0–1.0); therefore, these variables were not included in the final model. NON-EU-FOREIGNERS as additional predictor besides FOREIGNERS did not significantly improve the model.

We performed binary logistic regression using the following final model:

$${Logit\left(Lowacuity\right)}_{i}=\mathrm{log}{\left(\frac{Lowacuity}{1-Lowacuity}\right)}_{i}=\alpha +{\beta }_{1}{AGEGROUP}_{i}+{\beta }_{2}{GENDER}_{i}+{\beta }_{3}{SOCIALSTATUS}_{i}+{\beta }_{4}{WEEKEND}_{i}$$

Table 3 presents the results including ORs and 95%-confidence intervals (CIs). The odds for low-acuity were higher for young to medium age groups than for older age groups (60 +) and for females than for males. They were slightly but significantly higher for calls from a neighbourhood with lower social status and at the weekend. The results of goodness-of-fit tests were as follows: modified Hosmer–Lemeshow test for large samples: chi-squared = 79, *p*-value < 0.001. McFadden’s *R*^2^ = 0.008. *C*-statistic = 0.56. VIF < 1.1 for all predictors.

**Table 3:** Results of logistic regression analysis for a call being low-acuity, applied to the emergency call data of 2021

Variable	Adjusted OR (95% CI)	*p*-value
*Intercept*	0.37 [0.36–0.37]	*p* < 0.001 ***
*Age group*		
0–9	1.50 [1.45–1.55]	*p* < 0.001 ***
10–19	1.77 [1.71–1.83]	*p* < 0.001 ***
20–29	1.64 [1.59–1.68]	*p* < 0.001 ***
30–39	1.40 [1.37–1.44]	*p* < 0.001 ***
40–49	1.13 [1.10–1.16]	*p* < 0.001 ***
50–59	1.03 [1.00–1.05]	*p* < 0.05 *
60–69	1.00 [0.98–1.03]	*p* = 0.74
70–79	0.94 [0.92–0.96]	*p* < 0.001 ***
80–89 (reference)	1.0	
90 +	0.95 [0.92–0.99]	*p* < 0.05 *
*Gender*		
Male (reference)	1.0	
Female	1.12 [1.10–1.13]	*p* < 0.001 ***
*Social status index of the neighbourhood*	1.01 [1.00–1.01] per + 1 index unit increase (range from -1.5 to 4)	*p* < 0.05 *
*Weekend*		
No (reference)	1.0	
Yes	1.02 [1.00–1.04]	*p* < 0.05 *

Variables were not adjusted to other predictors than those in the model

^*^/*** Significance level was defined as *p* < 0.05 and marked with an asterisk (*). An even lower *p*-value of < 0.001 is marked with three asterisks (***)

## Discussion

One primary finding is that the increase in the number of emergency calls to the EMS Berlin was greater from 2018 to 2021 than the corresponding increase in population growth, whereas this is not specifically driven by low-acuity calls. Although we had no individual outcome data, i.e. no data on which diagnoses were later clinically confirmed on arrival at the patient or in the hospital or which patient ultimately survived the incident, our results are important evidence to provide a context for the recent media coverage [1, 2]. The increasing overall call number is underlined by official EMS operational statistics, showing that deployments increased by 8.7% from 2018 to 2021, both emergency rescues (+ 5.5%) and emergency transports (+ 37.7%) [40, 41]. Speculation about the reasons for the overall increase in utilisation is beyond the scope of this study. Although demographic ageing is a major issue in Germany, it is unlikely to be decisive here, because the population of Berlin is ageing slower than in many other parts of Germany, and its mean age remained constant at 42.6 years in the time period of the study (2018–2021) [42].

Among the low-acuity calls, some protocols and codes stand out because of their high absolute numbers (e.g. *abdominal pain with no complications* and *responsive person after fainting*).

The increasing protocol adherence is a result of MPDS quality management within the EMS, providing a nearly complete dataset for 2021. However, this raises the question of possible bias to the temporal analysis because it cannot completely be ruled out that some codes are missing not-at-random, when dispatchers aborted coding. The month-by-month analysis (Fig. 3) indicates that the calls with aborted protocols are in the majority high-acuity calls, because a rapid drop in protocol aborts within only 4 months is accompanied by a disproportionate increase in documented high-acuity cases, which is presumably a coding effect. However, there are three reasons why it is reasonable to assume that not *all* missing values are in reality high-acuity calls. Firstly, there is also a small increase in documented low-acuity cases within the 4 months. Secondly, internal quality management of the EMS Berlin indicates that some protocol aborts in earlier years was due to different training levels of individual dispatchers. Thirdly, some protocol aborts took place because until the beginning of 2020, call-taking without using full MPDS codes was accepted for the subgroup of calls which were transferred from other emergency call centres (e.g. public transport or police), and these cases presumably also include both low- and high-acuity cases. Taken together, it is reasonable to assume that the calls with unspecified acuity are in majority (nearly 90%) but not exclusively high-acuity calls. Only this extreme, hypothetical case would result in a very slight increase of the low-acuity proportion from 29.8 to 30%. Therefore, increasing protocol adherence did most likely not distort the low-acuity proportion to an extent that would reverse the acuity trend. The proportion of low-acuity calls remained more or less constant, it might have slightly decreased.

The regression sheds light on low-acuity predictors. Necessarily, the predictive power of the model in terms of goodness-of-fit is strongly limited, given that no data on individual pre-existing morbidity—which is decisive for whether a medical incident is per se acute—was available. Nevertheless, the regression reveals highly significant predictors. The most relevant variable is age, which should be interpreted in light of general morbidity. There is a certain bias due to the MPDS algorithms [43], because in some protocols older age leads to higher classifications in category and/or sub-determinant.

The social status index has an overall range of -1.5 (highest status) to 4 (lowest), but most neighbourhoods lie between -1 and 2. Comparing these values, the odds of low-acuity can be estimated to be 2.5% higher for the PLR with lower social status. It remains speculative whether this is caused by different availabilities of alternative health care resources, health literacy, morbidity or other effects. For example, it is possible that residents in areas with lower social status are less informed about suitable alternative health infrastructure and therefore call the EMS more often. It could also be that more non-life-threatening health incidents occur in areas with lower social status because pre-existing morbidity is higher. Future studies with additional data might further investigate this interesting correlation. Odds for a low-acuity call are 2% higher at the weekend, which could theoretically be due to less health care alternatives, but this also remains speculative.

The low-acuity odds were 12% higher for calls concerning females. Here, the different disease prevalence of women and men—especially in childbearing age, when low-code proportions are highest—should be kept in mind [44].

In this study, including the proportion of migrants did not noticeably improve the regression model. Berlin has a wide variety of migrant communities, with many people living in the city for a long time already, so the variable might be too imprecise to detect relevant differences in 112 calls.

### Geographical analysis

We found great differences in the age structure of patients between districts, which is logical given their different age composition, but only slight ones concerning low-acuity proportions.

For Bavaria, Hegenberg et al. found an increase of calls exceeding population growth [16], similar to the findings for Berlin. They demonstrated higher call rates in cities compared to rural municipalities and an association between municipality size and call proportion at weekends. Schehadat et al. analysed data from Rhineland-Palatinate regarding patient transport from the scene and concluded, despite some regional differences, that population density did not significantly determine EMS utilisation. In the present study, basic differences between more vs. less densely populated areas in Berlin were investigated. We observed a slightly different mean call number, which was not statistically significant.

### Low-acuity definition

We included all Omega and nearly all Alpha codes among the low-acuity codes. This is debatable and other classifications are justifiable. In principle, we agree with other researchers who have stressed that MPDS categories do not map the urgency of calls in a linear, symmetrical way [7, 11]. On the other hand, several studies show an association of MPDS codes with acuity and outcome to a certain degree: Garza et al. showed increasing rates of transportation with lights and sirens from the scene with increasing determinant level, and a < 1% rate for Alpha codes [8, 12, 43]. Hettinger et al. demonstrated a correlation between MPDS codes and ED admission vs. discharge [43]. Another US study detected only few inappropriate Alpha dispatches and concluded that MPDS has a good ability to identify higher-acuity patients when protocol adherence is good [8]. It could be argued to also consider selected Bravo or Charlie codes as low-code; however, it was decided, similar to the expert panel in a study by Shah et al. [6], not to include any (except for upcodings).

Not all low-acuity calls are suitable for handling without EMS involvement. However, they can be starting points for how to avoid overtriage [14] and how to find alternatives to ambulance dispatch, like OCMS visits, ambulatory appointments, mental health joint response car [45], Sociolance [46], advance provider response units [47] or pre-hospital emergency nurse [48]—depending on individual medical need and context. The EMS Berlin has recently expanded its code list for transfer to the OCMS [49], including many Alpha codes which are frequent in our analysis. For example, abdominal pain requires ED treatment only in a minority of cases, although these are not always trivial to identify [50]. The considerable number of upcodings (B00/C00) in the dataset indicates that the transferral is not always possible, at least partly due to OCMS capacity.

Omega codes—which are widely regarded as the lowest-acuity calls [11]—make up only for a small amount of what we defined as low-acuity. This suggests that Alpha codes play a major role in scenarios to optimise EMS resources.

### Implications for EMS organisation and health education

Given the increasing utilisation with a continuously high proportion of high-acuity cases and heterogeneous geographical distribution, the EMS should apply predictive dispatching in order to meet the challenging demand and secure quick responses. This means that available ambulances and other rescue infrastructure are allocated dynamically throughout the state of Berlin, estimating the most probable future demand in real-time.

Young people call the emergency number less frequently, but younger age is the strongest predictor for low-acuity. Therefore, this group might be suitable for target group-specific health education. An information campaign could inform them about alternative health care institutions, which are suitable for certain forms of low-acuity incidents and can improve the efficiency of care for all involved, because the patient does not need to wait for a long time in the ED, while the EMS and the ED save resources for life-threatening incidents.

Future studies should investigate the underlying morbidity of emergency calls and the predictors of cross-sectoral patient pathways after dispatch, which requires additional data. It would also be interesting to investigate the low-acuity call incidence depending on the spatial proximity of alternative medical infrastructure like hospitals and outpatient physicians. We recommend facilitating an international consensus on low-acuity MPDS codes, considering different national settings and emergency dispatch centres.

### Strengths and limitations

This is one of only few studies using a large individual-level secondary EMS dataset in Germany and the first to include all Berlin dispatch protocols from the recent four years. The study design benefits from MPDS implementation in Berlin as an internationally standardised system [26]. Entry errors can be considered as marginal due to the electronic documentation process. The study has good internal validity, as it provides a transparent, reproducible low-code classification.

A methodological limitation is the increasing MPDS protocol adherence (see the “Discussion” section). Predictive performance of the model is necessarily limited because no data on pre-existing individual morbidity is available. Sociodemographic indicators were available on regional, not on individual level. Therefore, each emergency call was merged with the sociodemographic parameter of the geographical area, which results in an approximation for each individual. This limits the accuracy of the regression compared to other variables like age which are available individually. We acknowledge this by referring to the geographical area when interpreting the results. We used the emergency locations as classifier for merging, which in some cases like commuters and tourists does not coincide with the place of residence. We found no direct evidence that the observed trends were specific for Berlin; however, the external validity with respect to other dispatch centres can be limited depending on different call-taking procedures and local health care context.

## Conclusions

This study of a large dataset covering four years provides important new insights into pre-hospital emergency care in Germany. The results do not support the hypothesis of minor cases and an ‘all-inclusive mentality’ being the primary driver of rising EMS demand. Overall utilisation increased, with a constant or slightly decreasing proportion of low-acuity calls. Young people call the emergency number less frequently, but younger age is the strongest predictor for low-acuity, so young people are a relevant group for future studies and for health literacy programmes.

## Data Availability

The call dataset is not publicly available and contains sensitive data. Most sensitive are the emergency locations which include street numbers. Therefore, special attention during anonymisation was paid to the transformation into LOR. Despite this, we cannot fully exclude that a person with special knowledge of an incident could re-identify single persons. Therefore, it is not possible to share this dataset with the public. However, the data may be obtained for further research directly from the fire department. The sociodemographic indicators used in this study are available from the Senate Department for Urban Development, Building and Housing Berlin [33, 34]. The datasets including population and geographical size of PGRs are available from the Office of Statistics Berlin-Brandenburg [35, 36].

## Abbreviations

BZR: District region
CI: Confidence interval
ED: Emergency department
EMS: Emergency Medical Service
ICREC: Imperial College Research Ethics Committee
LOR: Life-world-oriented region
MPDS: Medical Priority Dispatch System
NA: Not available/missing data
OCMS: On-Call Medical Service
OR: Odds ratio
PGR: Prognosis region
PLR: Planning region
UK: United Kingdom
US: United States of America
VIF: Variable inflation factor
